# Computational modeling of heterosynaptic plasticity in the hippocampus

**DOI:** 10.1186/1471-2202-16-S1-P1

**Published:** 2015-12-18

**Authors:** Peter Jedlicka, Lubica Benuskova, Wickliffe C Abraham

**Affiliations:** 1 Institute of Clinical Neuroanatomy, Neuroscience Center, Goethe University Frankfurt, Frankfurt am Main, Germany; 2 Department of Computer Science, University of Otago, Dunedin, New Zealand; 3 Department of Psychology University of Otago, Dunedin, New Zealand; 4 Brain Health Research Centre, University of Otago, Dunedin, New Zealand

## 

Hippocampal long-term potentiation (LTP) and long-term depression (LTD) are central synaptic mechanisms of learning and memory. Here we use compartmental models of hippocampal granule cells to better understand LTP and heterosynaptic LTD which have been reported in the dentate gyrus of awake rats [1]. Our simulations indicate that LTP and heterosynaptic LTD can be explained by a spike-timing-dependent plasticity (STDP) rule combined with a fast Bienenstock-Cooper-Munro (BCM)-like metaplasticity rule [2-5]. We study the interaction between these plasticity rules and ongoing pre- and postsynaptic activity. Our models are able to account for the experimentally observed degree of LTP and heterosynaptic LTD induced by various plasticity-inducing protocols.
